# The 21st Annual Meeting of the Rocky Mountain Virology Association

**DOI:** 10.3390/v13122392

**Published:** 2021-11-29

**Authors:** Laura A. St Clair, Ali L. Brehm, Shelby Cagle, Tillie Dunham, Jonathan Faris, Paul Gendler, Monica E. Graham, Sandra L. Quackenbush, Joel Rovnak, Rushika Perera

**Affiliations:** 1 Center for Vector-Borne Infectious Diseases, Department of Microbiology, Immunology, and Pathology, Colorado State University, Fort Collins, CO 80523, USA; stclairl@colostate.edu (L.A.S.C.); Ali.Brehm@colostate.edu (A.L.B.); Tillie.Dunham@rams.colostate.edu (T.D.); 2 Department of Microbiology, Immunology, and Pathology, Colorado State University, Fort Collins, CO 80523, USA; Shelby.Cagle@colostate.edu (S.C.); Sandra.Quackenbush@colostate.edu (S.L.Q.); Joel.Rovnak@colostate.edu (J.R.); 3 Department of Chemical and Biological Engineering, University of Colorado Boulder, Boulder, CO 80309, USA; Jonathan.Faris@colorado.edu; 4 Department of Molecular, Cellular, and Development Biology, University of Colorado Boulder, Boulder, CO 80309, USA; Paul.Gendler@colorado.edu; 5 Department of Immunology & Microbiology, University of Colorado Anschutz Medical Campus, Aurora, CO 80045, USA; monica.graham@cuanschutz.edu

**Keywords:** flavivirus, SARS-CoV-2, pandemic preparedness, host–virus interactions, prion, vaccines, interferon lambda, virus

## Abstract

Nestled within the Rocky Mountain National Forest, 114 scientists and students gathered at Colorado State University’s Mountain Campus for this year’s 21st annual Rocky Mountain National Virology Association meeting. This 3-day retreat consisted of 31 talks and 30 poster presentations discussing advances in research pertaining to viral and prion diseases. The keynote address provided a timely discussion on zoonotic coronaviruses, lessons learned, and the path forward towards predicting, preparing, and preventing future viral disease outbreaks. Other invited speakers discussed advances in SARS-CoV-2 surveillance, molecular interactions involved in flavivirus genome assembly, evaluation of ethnomedicines for their efficacy against infectious diseases, multi-omic analyses to define risk factors associated with long COVID, the role that interferon lambda plays in control of viral pathogenesis, cell-fusion-dependent pathogenesis of varicella zoster virus, and advances in the development of a vaccine platform against prion diseases. On behalf of the Rocky Mountain Virology Association, this report summarizes select presentations.

## 1. Introduction

In 2000, the Rocky Mountain Virology Club (RMVC) was formed to create an environment where regional scientists could showcase their recent advances in virology, graduate students could interact with senior scientists, and productive collaborations could flourish among graduate students, postdocs, junior investigators, and senior scientists across our region. In 2009, we added prion biologists and investigators to our group, and in 2010, RMVC became the Rocky Mountain Virology Association (RMVA). For most of our gatherings, we have held the annual RMVA meeting at the Colorado State University Mountain Campus located ~50 miles west of the main CSU campus in Fort Collins, CO. In 2020, our annual gathering was held virtually, like many other conferences, due to the ongoing coronavirus pandemic. We were determined to ensure that this year’s meeting could once again be held in person. To that end, we required that all attendees provide proof of vaccination and a negative COVID-19 test taken with 48 h of the conference. We also wore masks and practiced social distancing as much as possible. Happily, we report that there have been no reported instances of COVID-19 infections amongst our participants following this year’s gathering. Taking these safety precautions allowed all of us to enjoy a much-needed, secluded retreat in the beautiful Colorado Rocky Mountains, and to reconnect (or form new connections) with friends, colleagues, and mentors over our shared love of viruses and prions.

**Figure 1:**
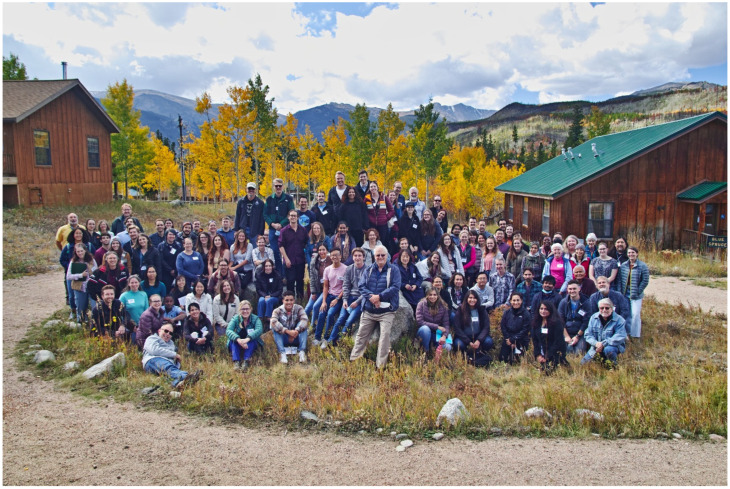
Attendees at the 21st Annual Rocky Mountain Virology Association Conference.

**Figure 2:**
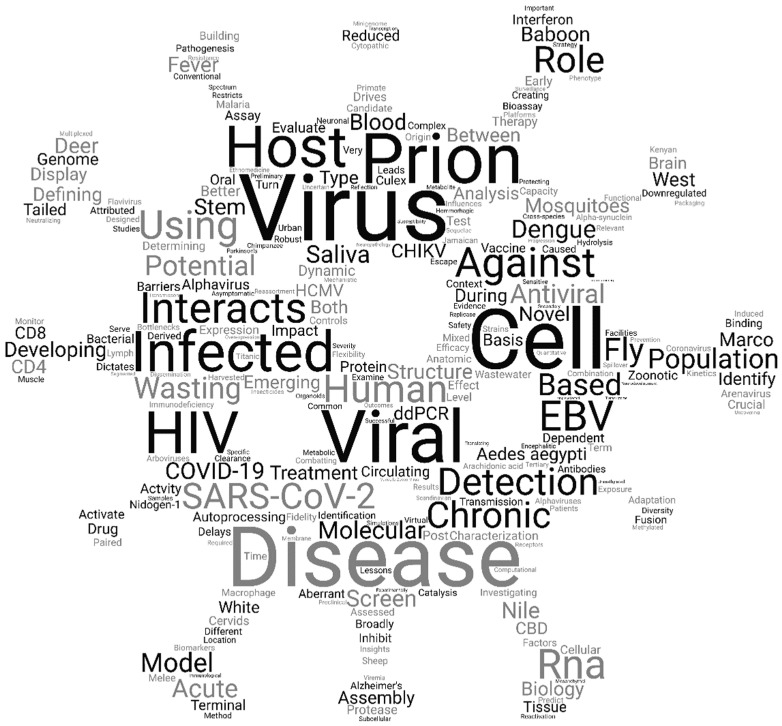
The Longest Coherent Sentence Word Cloud and Award. Brynn Lauterbach of Colorado State University won with her sentence of 109 words: “Surveillance of exposure, asymptomatic transmission, dissemination, activity, progression, and antiviral drug resistance of important emerging cross-species viral disease of humans, urban *Aedes aegypti* and *Culex* mosquitoes, Scandinavian sheep, white tailed deer, Jamaican baboons, Kenyan chimpanzees, cervids, and specific relevant primates in different locations impacts the detection of arbovirus, flavivirus, arenavirus, and alphaviruses and is crucial in determining the level of host spillover and developing a cellular or RNA vaccine, stem cell-derived therapy, circulating neutralizing antibodies, and creating complex and sensitive quantitative immunological bioassays, novel computational model simulations, and ddPCR in experimentally assessing and characterizing expression of cellular factors, biomarkers, and susceptibility of samples against a spectrum of combination treatments.” The word cloud was generated using the WordArt.com word cloud generator, and image licensing was provided to Laura A. St. Clair.

This year’s three-day gathering was attended by 114 participants made up of undergraduate students, graduate students, postdocs, research scientists, and junior and senior faculty members (Figure 1). We enjoyed 31 oral presentations and 30 poster presentations, including the keynote address provided by Dr. Ralph Baric and talks from our invited speakers: Drs. Nicole Ehrhart, Joyce Jose, Rupika Delgoda, James Heath, Helen Lazear, Stefan Oliver, and Holger Wille. Talks ranged from advances made in SARS-CoV-2 surveillance, detection, and vaccine development; characterization and analyses of potential emerging zoonoses; molecular determinants of viral replication and pathogenesis; and advances in prion biology that have culminated in advanced understanding of prion disease dissemination and development of a prion vaccine platform. For a bit of levity, students also participated in the “Longest Coherent Sentence” contest, using a word cloud comprised of all the words from the abstracts presented at this year’s conference. The winner, Brynn Lauterbach of Colorado State University, took home the prize with her 109-word sentence (Figure 2).

**Figure 3:**
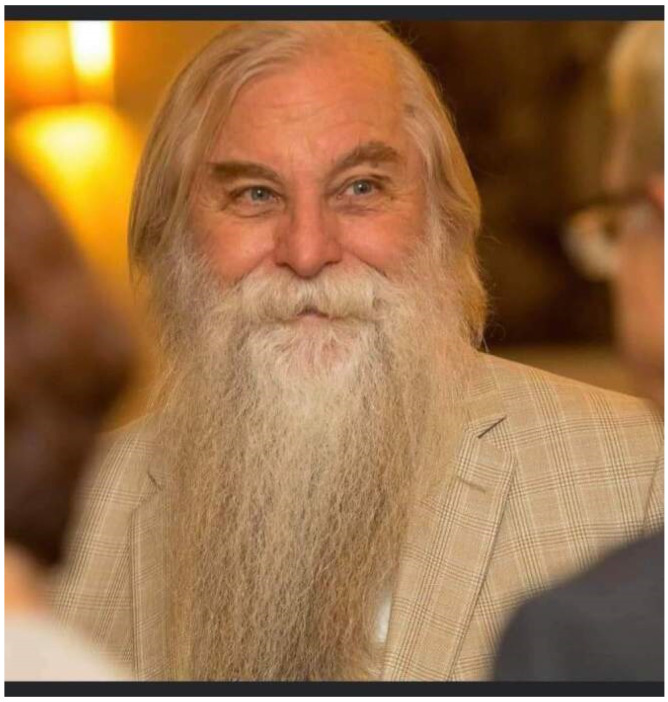
Dr. Randall J. Cohrs. Photo permitted for use by the Cohrs family.

However, this year’s gathering was also bittersweet for many of us in attendance, as our dear friend, colleague, founding member, and president of RMVA, Dr. Randall J. Cohrs (Figure 3), passed away suddenly this past July. To say that Randy was a cornerstone of the RMVA conference and within our virology community is an understatement, and his absence was assuredly felt at this year’s conference. Professor Emerita Dr. Kathryn V. Holmes of the University of Colorado School of Medicine, who was unable to join us physically, provided a poignant video tribute to Randy that highlighted his years of service and dedication to the virology community. Randy loved a good story, but especially those that unraveled the inner workings of viral and prion diseases. He poured that love into the formation of innumerable global collaborations, and in the formation of regional, national, and international conferences where knowledge can be shared, and training and mentorship could be extended to students and junior scientists. His kindness, generosity, friendship, mentorship, and perpetual childlike joy for science are greatly missed, but were a blessing to all of us that knew him. To honor his legacy, the Randall J. Cohrs Awards Fund was established this year to support the top student and postdoc poster and presentation awards and to support the Randall J. Cohrs Keynote address. We would also like to dedicate this year’s conference report to his memory. Selected abstracts are presented below. 

## 2. Summary of Scientific Sessions

### 2.1. Keynote Speaker

Dr. Ralph Baric (Department of Microbiology and Immunology, William R. Kenan, Jr. Distinguished Professor, Department of Epidemiology, University of North Carolina School of Medicine, Chapel Hill, NC, USA) presented the keynote address and discussed emerging coronavirus pathogenesis and prevention. Emerging viruses constantly arise from zoonotic reservoirs to threaten global health and economic stability, illustrated by the ongoing SARS-CoV2 COVID-19 pandemic that has infected over 200 million people and caused over 4.6 million deaths worldwide. Zoonotic coronaviruses include rich pools of strains poised for human emergence, as evidenced by their ability to use human receptors for docking and entry, replicate efficiently in primary human airway epithelial cells, and cause disease in transgenic mice. Using well-developed reverse genetic and animal models of human disease, his group explores the evolutionary and pathogenic potential of SARS-CoV-2 and other emerging coronaviruses that are poised to cause acute and chronic disease, discuss mechanisms of severe acute and chronic lung injury, and explore new strategies for antiviral control and vaccine design. An underlying theme is that human globalization, behavior, and activities will increase the probability of epidemic and pandemic viral disease outbreaks in the 21st century, requiring new investments and strategies for preparedness at the national and international levels.

### 2.2. Prions and Protein Aggregation

Brianne Coleman (Department of Microbiology, Immunology, and Pathology, Colorado State University, Fort Collins, CO, USA) updated on her work on detecting prions in specific blood cell populations harvested from pre-clinical white-tailed deer infected with chronic wasting disease. Chronic wasting disease (CWD) is a terminal, infectious prion disease endemic within captive and free- ranging cervid populations across North America, Korea, and Scandinavia. Big-game hunting (1 in 36 Americans) and subsequent human consumption of contaminated meat (7000–15,000 CWD+ cervids/year; rising 20%/year) intensifies the need to characterize prion peripheralization in cervids. It has been established that prion infectivity is present in the blood of prion-infected animals, including deer and humans. Yet to be revealed is the longitudinal profile of specific blood cell subsets associated with prion infection. Using her white-tailed deer model, she has established a reliable, consistent method for isolating blood cell populations throughout the disease course—from minutes post-inoculation to terminal disease. Throughout the CWD disease course, she has isolated platelets, polymorphonuclear cells (PMNs), total peripheral blood mononuclear cells (PBMCs), and specific blood cell subsets, including CD4, CD8, and CD14. All of these cell subsets are being analyzed for the presence of amyloid seeding activity (prions) with real-time quaking-induced conversion (RT-QuIC) assays. To date, she has analyzed total PBMC and specific blood cell subsets with RT-QuIC and has identified the presence of amyloid seeding activity near the time of tonsil biopsy positivity. Longitudinal analysis of these cell populations is ongoing. Results from these studies will identify the role specific blood cell subsets play in establishing and maintaining CWD prion infections and may offer early prion antemortem diagnosis. All animal studies were performed following guidelines and protocols approved by the Institutional Animal Care and Use Committee of Colorado State University.

Vincenzo Gilberto, along with Stephanie McGrath and Julie A. Moreno (Department of Environmental and Radiological Health Sciences and Department of Clinical Sciences, Colorado State University, Fort Collins, CO, USA), presented his work on neuronal treatment of models of Alzheimer’s disease using CBD and trazodone. Alzheimer’s disease (AD) is one of the most common forms of neurodegeneration and is typically characterized by either the presence of Amyloid-Beta plaques (Aβ) or Tau Tangles (NFT). Even though there is no current cure for AD, it is well known that targeting of signaling pathways involved in reactive oxygen species (ROS) or unfolded protein response (UPR) improves behavioral deficits, glial inflammation, and neuronal toxicity. This research aims to utilize specific strains from the model organism *C. elegans* that have been genetically modified to contain two common misfolded proteins found to aggregate and accumulate in AD patients’ brains, amyloid-β, and the hyperphosphorylation of tau (P-tau). They hypothesize that combinational drug stacking of compounds that target both ROS production and UPR will improve the behavioral qualities associated with AD and will also extend the lifespan of these neurodegenerative nematode models to that of their control counterparts. To address this hypothesis, they have used CBD and trazodone to inhibit ROS and the UPR, respectively. Using both one-way and two-way ANOVA, their previous experiments have revealed that neurodegenerative *C. elegans* motility is significantly worse compared to their control counterparts and that early-stage exposure to trazodone significantly improved the motility of the neurodegenerative nematodes. The next steps for this project include, but are not limited to, isolated and combinational drug treatments of both trazodone and CBD to measure their motility and life span. Once optimal doses are identified, they aim to implement a late-stage rescue of neurodegenerative *C. elegans* with combinational drug stacking. All animal studies were performed following guidelines and protocols approved by the Institutional Animal Care and Use Committee of Colorado State University.

Caitlyn Kraft, along with C. Hoover, N. Denkers, and Candace Mathiason (Department of Microbiology, Immunology, Colorado State University, Fort Collins, CO, USA), presented her work on detection of chronic wasting disease prions in muscle tissue. Prion diseases affecting animals that are regularly consumed by humans pose a significant public health risk—the most notable example being bovine spongiform encephalopathy, or Mad Cow Disease, which spread to humans through consumption of infected muscle. Chronic wasting disease (CWD) affects moose, elk, and deer, all of which are commonly hunted and consumed by humans. While clear evidence of CWD transmission to humans has not been demonstrated, establishing whether CWD prions are present in muscle is of high interest. Currently, conventional assays, such as immunohistochemistry (IHC) and enzyme-linked immunosorbent assay (ELISA), are unable to detect the low concentration of prions likely to be found outside neural or lymphatic tissues. Here, they combined two prion amplification assays—protein misfolding cyclic amplification (PMCA) and real-time quaking-induced conversion (RT-QuIC)—to demonstrate the presence of prion seeding activity in the muscle of CWD-positive white-tailed deer. Hamstring muscle samples from 24 CWD-infected deer were subjected to five rounds of PMCA, followed by a readout of the amplification product with RT-QuIC. They demonstrated that 16 of the 24 samples (66.67%) contained prion seeding activity. To compare the sensitivity of PMCA and RT-QuIC, a subset of muscle samples were examined with IHC and standard RT-QuIC. Zero (0) of the four samples assessed with IHC and one of the four samples assessed with RT-QuIC tested positive. They concluded that the combination of prion amplification assays allowed for the detection of low prion concentrations present in the muscle of CWD-positive deer. The consequence of consumption of these low levels of CWD prions remains unknown. All animal studies were performed following guidelines and protocols approved by the Institutional Animal Care and Use Committee of Colorado State University.

Casey P. McDermott ^1^, along with Collin Bantle ^1^, Dev Aldaz ^2^, Savannah Rocha ^2^, Katriana Popichak ^2^, Ken Olson ^2^, and Ronald Tjalkens ^1^ (^1^ Toxicology Program, Dept. of Environmental and Radiological Health Sciences, ^2^ Dept. of Microbiology, Immunology, and Pathology, Colorado State University, Fort Collins, CO, USA), examined how alpha-synuclein overexpression delays neuropathology in a viral model of Parkinson’s disease. Parkinson’s disease (PD) is a multifaceted movement disorder caused by the interaction of multiple factors, including age, environmental exposures, and genetic predisposition. The hallmarks of PD include glial reactivity, neuronal loss in the Substantia Nigra (the movement center of the brain), loss of dopamine in the striatum, and alpha synuclein aggregation. Infection with Western Equine Encephalitic Virus (WEEV) in animals and humans has previously been shown to cause neurological symptoms similar to those of PD. In this study, they examined how WEEV could propagate neuropathology in transgenic mice expressing a variant of human alpha-synuclein (A53T) associated with early onset of PD. Normally, these transgenic mice do not show any motor deficits, α-syn aggregation, or neuronal loss until 16 months of age. They postulate that infection with WEEV would accelerate aggregation of A53T alpha-synuclein at as early as 4 months of age (1 month post infection). To test this hypothesis, mice were intranasally infected with WEEV at 3 months of age and examined for neuropathological changes at 1 and 3 months post-infection. Cellular markers of neuropathology were assessed in multiple brain regions by immunohistochemistry and immunofluorescence staining, including glial reactivity, protein aggregation, and neuronal loss. At 1 month post-infection, they saw extensive astrogliosis in both the Substantia Nigra and the striatum. There was a significant increase in microgliosis in the Nigra as well; however, there was no loss of dopaminergic neurons. Total α-syn was also measured in all regions, and was only found to be significant in the cortex. Three months post-infection, microgliosis and astrogliosis had ceased, and they began to see loss of TH neurons. Alpha-synuclein aggregation also began to rise in the SN and the cortex. This supports the field in such that α-syn could have an antiviral application, even in a form that should be pre-disposed to misfolding.

Julianna Sun ^1,2^, along with Jifeng Bian^1^, Sehun Kim^1^, Jenna Crowell ^1^, Bailey Huser ^1^, EmmaKate Raisley ^1^, and Glenn C. Telling^1,2^ (^1^ Prion Research Center and the Department of Microbiology, Immunology, and Pathology, Colorado State University, ^2^ Program in Cell and Molecular Biology, Colorado State University, Fort Collins, CO, USA), presented her work detailing the breadth, properties, and zoonotic potential of emergent prion disease strains in Scandinavian cervids. The replicative properties of prions, which are extraordinary proteinaceous infectious agents causing lethal neurodegenerative diseases in humans and animals, challenge fundamental concepts of inheritance and infection. During this process, PrPSc, a conformationally altered counterpart of host-encoded PrPC, templates its infective conformation on PrPC, resulting in exponential prion accumulation. Remarkably, prions share the property of heritable strain diversity with nucleic-acid-containing pathogens. Transmission of bovine spongiform encephalopathy to humans epitomizes the unpredictable effects of strains on the prion host range and the devastating effects of prion zoonoses. Chronic wasting disease (CWD), a burgeoning and ineradicable disease of wild and captive North American (NA) cervids, raises similar concerns. The discovery of CWD in Norway in 2016 raised questions about its relationship to the NA epidemic. Their finding that emergent Norwegian cases are, in fact, caused by novel prion strains with unpredictable adaptive potential increases uncertainties about CWD zoonosis. The development of genetically modified mice has been key for advancing our understanding of the molecular parameters controlling prion transmission, including the effects of species-specific variation in PrP primary structures. While deer express glutamine (Q) at PrP residue 226, elk express aspartate (E). To investigate the role of residue 226 on strain selection, they developed gene-targeted mice in which murine PrP was replaced by elk or deer PrP, referred to as GtE or GtQ, respectively. They showed that residue 226 controls the selective propagation of distinct CWD strains and that the influences of residue 226 differ between Norwegian and NA CWD strains. Additional CWD cases have been diagnosed in Norway, Sweden, and Finland. Here, they described the results of studies that illustrate additional unprecedented strain properties of these CWD isolates and described approaches to addressing the risks they pose to sympatric species and humans exposed to CWD. This work was supported by NINDS grants 1R01NS109376 and 1R01NS121682. All animal studies were performed following guidelines and protocols approved by the Institutional Animal Care and Use Committee of Colorado State University.

Holger Wille (Centre for Prions and Protein Folding Diseases and Department of Biochemistry, University of Alberta, Canada) presented his and his colleagues’ efforts to design prion disease vaccines. Chronic wasting disease (CWD) is a prion disease that affects captive and free-ranging cervids in many U.S. states and Canadian provinces, including Colorado and neighboring states. CWD is spreading uncontrollably through environmental contamination and animal-to-animal contacts. The prion diseases are caused by the misfolding of the cellular prion protein (PrPC), which adopts a β-sheet rich conformation when it is converted into the infectious state (PrPSc). Prion protein peptides have been used in previous attempts to create prion vaccines, but in most cases, the structures of the antigens were poorly controlled, resulting in a lack of vaccine efficacy. Here, he presented their approach to designing structure-based vaccines that present specific antigenic sequences in a structurally controlled format, thereby creating a prophylactic prion vaccine. The prion domain of the fungal HET-s protein is unrelated to the mammalian prion protein, but is thought to adopt a similar, β-sheet rich conformation. This innocuous scaffold protein was engineered as a vaccine carrier to express PrPSc surface epitopes in a structurally controlled manner. They selected seven discontinuous amino acids, which were predicted to be surface exposed in PrPSc, and inserted them into equivalent positions in the scaffold protein. The vaccine was produced in *E. coli*, purified, refolded, controlled for its structural fidelity, and used to immunize a transgenic mouse model (TgP101L mice) of a genetic prion disease in humans: Gerstmann–Sträussler–Scheinker disease. Their structure-based vaccine targeting PrPSc produced an immune response that was specific for the infectious conformer only and did not react with PrPC. This specificity for PrPSc was observed in all animals that were immunized with the vaccine, while animals that received the unmodified scaffold protein produced only unspecific immune responses. Moreover, unimmunized TgP101L mice developed disease symptoms at ~177 days of age (±17 days), animals that were immunized with the unmodified scaffold protein started to fall ill at ~166 days of age (±26 days), while TgP101L mice that were immunized with the prion vaccine remained free of symptoms until ~448 days of age (±39 days). Therefore, their prion vaccine extended the health span of the TgP101L mice by >250%. Additional vaccine efficacy trials in other prion disease models are currently underway. Furthermore, a prophylactic vaccine efficacy trial in CWD-challenged white-tailed deer will be initiated shortly in collaboration with Candace Mathiason and her team at Colorado State University. The project was supported by awards from the Alberta Prion Research Institute, the Alberta Livestock and Meat Agency, and the Creutzfeldt-Jakob Disease Foundation. All animal studies were performed following guidelines and protocols approved by the Institutional Animal Care and Use Committee of the University of Alberta. All studies using human subjects or tissue samples have been approved by the Institutional Review Board of the University of Alberta.

### 2.3. Arboviruses

Stephanie E. Ander ^1^, along with Kathryn S. Carpentier ^1^ and Thomas E. Morrison ^1^ (^1^ Department of Immunology and Microbiology, University of Colorado School of Medicine), presented on their work on the magnitude and duration of vertebrate viremia. These parameters are critical determinants of arbovirus transmission, geographic spread, and disease severity. However, mechanisms determining arboviral viremia levels are poorly defined. Their laboratory previously defined roles for liver Kupffer cells and scavenger receptor SR-A6 (MARCO) in mediating control of arthritogenic alphavirus viremia. To determine whether this is specific for arthritogenic alphaviruses or functions more broadly, they evaluated the clearance of Eastern (EEEV) and Venezuelan (VEEV) equine encephalitis viruses using recombinant virus particles composed of a chimeric SINV-EEEV or -VEEV genome encapsulated by EEEV or VEEV structural proteins. Following intravenous inoculation of mice, they found that EEEV^FL93^ and enzootic VEEV^PIXV^ particles are cleared from circulation by Cd169^+^ phagocytic cells, while epizootic VEEV^TrD^ particles are resistant and persist at >3 hpi within the bloodstream. Furthermore, EEEV^FL93^ clearance is dictated by the presence of a basic patch on the E2 glycoprotein. Alanine substitution of these residues not only produces clearance-resistant EEEV^FL93^ particles, but has also been shown to enhance viremia and viral pathogenesis following subcutaneous inoculation. While similar, the specific mechanisms mediating vascular clearance of arthritogenic and encephalitic alphaviruses are distinct: EEEV^FL93^ and VEEV^PIXV^ vascular clearances have slower kinetics and are MARCO-independent. Collectively, their findings suggest that phagocytic cells control the magnitude and duration of viremia following infection with a broad group of alphaviruses. Ongoing studies are aimed at defining the specific phagocytic cells and receptors that mediate clearance of circulating EEEV^FL93^ and VEEV^PIXV^ and the molecular features of EEEV and VEEV particles that promote or evade vascular clearance. All animal studies were performed following guidelines and protocols approved by the Institutional Animal Care and Use Committee of The University of Colorado Anschutz Medical Campus.

Anna Burnet (Department of Microbiology, Immunology, and Pathology, University of Colorado Anschutz, Boulder, CO, USA) discussed her efforts in defining a role for heme in reactivation of EBV from latency in B cells during acute malaria. The connection between Epstein–Barr virus (EBV) and *Plasmodium falciparum* malaria in their role in the development of EBV-associated Burkitt lymphoma in Sub-Saharan Africa has long been accepted by the scientific community, yet the mechanisms involved remain unknown. EBV-positive Burkitt lymphoma is a non-Hodgkin’s lymphoma with peak incidence at 6 years of age. A major hallmark of malarial disease is hemolysis of red blood cells, which causes the release of heme in large quantities. She found that hemin treatment of latently infected EBV-positive B cells in culture results in viral reactivation from latency by activating transcription of immediate early genes BZLF1 and BRLF1, as well as degradation of Bach2 protein by 24 h. Hemin treatment additionally increases CD138 expression, demonstrating that cells are being driven to shift to plasma cells. She hypothesized that heme released during acute malaria infection drives differentiation of latently infected EBV-positive memory B cells via specific heme-binding to Bach2, an important regulator of B cell proliferation and differentiation. Importantly, Bach2 is a repressor of Blimp-1, a protein essential for B-cell terminal differentiation. Furthermore, she hypothesized that the binding interaction between heme and Bach2 can allow for an increase in Blimp-1, which, in turn, can induce lytic replication by initiating transcription of immediate early viral genes BZLF1 and BRLF1. No animal or human studies were performed.

Jasmine Donkoh (Department of Microbiology, Immunology and Pathology, Colorado State University, Fort Collins, CO, USA) presented on her work studying the effect of dengue virus infection on macrophage gene expression and phenotype. Dengue virus (DENV) is the most prevalent arthropod-borne flavivirus in the world, causing dengue hemorrhagic fever and dengue shock syndrome. Macrophages are a site of DENV replication, and the imbalance between macrophage pro-inflammatory and anti-inflammatory phenotypes during DENV infection leads to severe disease outcomes. To investigate the effect of macrophage phenotype on DENV serotype 2 (DENV2) replication, she treated THP-1 macrophages with either interferon gamma (IFNγ) or interleukin 4 (IL-4) to polarize them towards pro-inflammatory or anti-inflammatory phenotypes, respectively, prior to infection. DENV2 showed preferential replication in cells treated with IL-4 compared to cells treated with IFNγ or non-treated cells. DENV2 infection upregulates expression of the anti-inflammatory cytokine, interleukin 10 (IL-10), and the inflammatory chemokine, CXCL10, in all phenotypes. DENV2 infection of macrophages also leads to induced expression of the host mediator complex protein, cyclin dependent kinase 8 (CDK8). CDK8 is a transcriptional co-factor that regulates expression of IFNγ-stimulated genes and certain cytokines/chemokines. Expression of CDK8 increases coincidently with DENV2 replication throughout a 36-h time course. Treatment with Senexin B, a CDK8 inhibitor, decreased DENV2 mRNA and infectious particles in THP-1 macrophages. She also found that inhibition of CDK8 activity with Senexin B increases IL-10, but decreases CXCL10 gene expression, regardless of macrophage phenotype. This suggests dependence upon CDK8 activity for virus induction of CXCL10 and IL-10. She hypothesizes that CDK8 plays a key role in regulating a macrophage’s ability to transcribe genes that control polarization and antiviral immunity. No animal or human studies were performed.

Tillie Dunham, along with Mark Stenglein (Department of Microbiology, Immunology and Pathology, Colorado State University, Fort Collins, CO, USA), discussed vertical transmission of insect-specific viruses in *Aedes Aegypti* mosquitos. *Aedes Aegypti* mosquitoes are a major vector of pathogenic arboviruses, including the dengue and zika viruses. In this project, she quantified the vertical transmission efficiency of insect-specific viruses that are common in wild mosquitoes. These viruses cannot be transmitted to vertebrates, but may impact the biology of their host. Viruses with efficient vertical transmission have an inherent ability to spread through host populations and so could have important applications in biocontrol. She first reared mosquitoes from eggs, crossed them, and sampled adults and offspring from those crosses. Then, she performed RNA extraction and RT and qPCR in order to screen for viruses in each sample. She found that three viruses, Verdadero virus, Renna virus, and *Aedes* anphevirus, exhibited efficient biparent vertical transmission. This property likely underlies their success in the wild and their persistent infection of colonized mosquitoes and means that these could be good candidates for gene delivery through mosquito populations. No animal or human studies were performed.

Emily Fitzmeyer, along with Emily N. Gallichotte, Nicole R. Sexton, and Gregory D. Ebel (Department of Microbiology, Immunology, and Pathology, Colorado State University, Fort Collins, CO, USA), presented her efforts using barcoded West Nile virus to examine the impact of tissue and cellular bottlenecks on virus populations in *Culex* mosquitoes. Due to error-prone replication, RNA viruses exist within hosts as genetically and phenotypically complex swarms. For arboviruses such as West Nile virus (WNV), these swarms fluctuate as they encounter bottlenecks and different selective environments when cycling between hosts. Bottlenecks alter the composition of virus populations by stochastically reducing overall diversity, which can impact virus fitness and transmission potential. Using a molecularly barcoded WNV (bcWNV) allows them to quantitatively measure population bottlenecks in two common WNV mosquito vectors: *Culex tarsalis* and *Culex quinquefasciatus*. This approach allows them to examine changes in virus population structure as the population moves through the midgut, salivary glands, and saliva—the main bottlenecks associated with infection, dissemination, and transmission. Additionally, it is not currently known which cell populations in the midgut and salivary glands become infected and what role intracellular bottlenecks might play in shaping virus populations. Previous work with barcoded viruses shows that single cells may be infected with several unique WNV genomes (i.e., become polyinfected). They therefore will use single-cell sorting and sequencing of bcWNV-infected mosquito tissues to determine the number of viral barcodes present in both whole tissues and specific cell populations within these tissues. While it is known that there are bottlenecks at the tissue level, they predict that interactions at the cellular level are mainly responsible for altering the diversity of the total virus population. From this work, they will establish how both whole-tissue and intracellular bottlenecks alter virus populations within mosquitos. This work was supported by NIH grant R01 AI067380. No animal or human studies were performed.

Monica Graham ^1^, Benjamin Akiyama ^2^, and David Beckham ^1^ (^1^ Department of Immunology and Microbiology, University of Colorado Anschutz Medical Campus, ^2^ Department of Biochemistry and Molecular Genetics, University of Colorado Anschutz Medical Campus, Aurora, CO, USA) discussed the role of secondary and tertiary structures within the 3′ untranslated region (UTR) of Zika virus (ZIKV) in viral cytopathic effects. The dumbbell-1 (DB-1) structure is one of the least-studied RNA structures in the 3′UTR. Previous studies suggest that DB-1 in ZIKV and other flaviviruses is important for genome replication and cytopathic effect (CPE). However, these studies have not investigated how the highly conserved DB-1 structure is related to its function. To investigate the structure–function relationship of DB-1, they created two mutant infectious ZIKV clones: TL.PK, which disrupts a crucial tertiary fold, and p.2.5′, which disrupts the conserved secondary structure. In A549 cells, viral genome replication is modestly reduced, but the CPE of both mutants is significantly abrogated. They investigated sub-genomic flaviviral RNA (sfRNA) formation with their TL.PK and p.2.5′ mutants during A549 infection and found that their mutants produced less sfRNA compared to ZIKV-WT. The two mutants also did not produce the smallest sfRNA species seen in ZIKV-WT, sfRNA3. To investigate the decreased CPE, they assayed mutant-infected cells for cell viability and caspase induction. They found that cell viability was significantly increased in TL.PK- and p.2.5′-infected cells compared to ZIKV-WT. It was also determined that TL.PK- and p.2.5′-infected cells had decreased levels of activated caspases 1 and 3 compared to ZIKV-WT. Overall, these data suggest that the DB-1 structure plays an important role in ZIKV CPE and sfRNA formation during mammalian cell infection. Their current hypothesis is that the phenotypic change in CPE is a result of altered sfRNA production during infection. No animal or human studies were performed.

Shannon N. Hinsdale, along with Kathryn S. Carpentier and Thomas E. Morrison (Department of Immunology and Microbiology, University of Colorado School of Medicine, Aurora, Colorado, USA), discussed the development of an shRNA-based screen to identify ISGs with antiviral activity against type I interferon sensitive alphaviruses. Alphaviruses are positive-sense RNA arboviruses that can cause severe human disease. Alphavirus infection elicits and is sensitive to type I IFN. Type I IFN signaling transcriptionally activates antiviral interferon-stimulated genes (ISGs). However, specific ISGs that control alphavirus infection remain poorly characterized. Here, Ross River virus (RRV) is used as a representative alphavirus to identify ISGs that control alphavirus replication. RRV DC5692 is an attenuated strain of RRV with enhanced sensitivity to type I IFN both in vitro and in vivo compared with RRV T48. This phenotype was attributed to six nonsynonymous nucleotide changes in nsP1. They developed a flow-cytometry-based ISG knockdown screen by utilizing lentiviruses that co-express GFP and shRNA targeting ISGs and an mKate-expressing RRV encoding the six nsP1 mutations to identify ISGs that control alphavirus replication. Murine embryonic fibroblasts (MEFs) were chosen for the screen after comparing transduction efficiency in L929 cells and MEFs via flow cytometry. Virus MOI (FFU/cell) and type I IFN treatment conditions were optimized by pre-treating WT or transduced MEFs with a range of type I IFN concentrations and infecting with a range of MOIs. An MOI of 3 was determined to be sufficient for infection of ~80% of cells and a concentration of 7.5 IU/mL type I IFN suppressed the number of mKate-positive cells by more than 10-fold. Using these conditions, they anticipate that this screen will identify specific ISGs that control alphavirus replication and are antagonized by determinants in nsP1, thus improving the understanding of alphavirus pathogenesis.

Helen Lazear (Department of Microbiology and Immunology, University of North Carolina Chapel Hill, Chapel Hill, NC, USA) presented her lab’s work investigating how interferon lambda (IFN-λ, type III IFN) restricts viral infection at anatomic barriers. Crossing anatomic barriers is a key step in viral infection, and the ability to cross external epithelial barriers (such as the respiratory and gastrointestinal tracts or the skin) and internal barriers (such as the blood–brain barrier or placenta) contributes to viral tissue tropism and transmission mechanisms. As a mosquito-borne virus, Zika virus (ZIKV) must first surmount the external barrier of the skin, and its ability to cross the placental barrier allows ZIKV to cause birth defects. Among the specialized immune mechanisms that protect anatomic barriers, IFN-λ provides front-line protection by activating a local antiviral response similar to IFN-αβ (type I IFN), but with reduced inflammation. IFN-λ is constitutively secreted from human placental cells and reduces ZIKV transplacental transmission in mice, but the mechanism by which it restricts congenital infection is unknown. They generated mouse pregnancies that lacked either maternal or fetal IFN-λ responsiveness and found that IFN-λ signals exclusively to maternal tissues to exert antiviral effects against ZIKV. Interestingly, infection earlier in pregnancy (E7 rather than E9) resulted in IFN-λ-mediated pathology: IFN-λ-responsive dams had higher rates of fetal resorption than non-responsive dams. Maternal IFN-λ-mediated pathology was also elicited by poly(I:C) treatment, which was similarly dependent on gestational stage. These findings identify an unexpected effect of IFN-λ signaling specifically in maternal (rather than placental or fetal) tissues and highlight the complexity of immune signaling at the maternal–fetal interface, where disparate outcomes can result from signaling at different gestational stages. While IFN-λ does not restrict replication of ZIKV in peripheral tissues, they found a skin-specific role for IFN-λ in restricting ZIKV dissemination. To further investigate the antiviral effects of IFN-λ in the skin, they used a herpes simplex virus (HSV-1) skin infection model. They found that mice lacking IFN-λ signaling developed more severe skin lesions compared to WT mice, identifying a protective role for IFN-λ in the skin. Altogether, this work highlights the importance of IFN-λ-mediated immunity at both external and internal barriers for controlling viral pathogenesis. This work was supported by R01 AI139512. All animal studies were performed following guidelines and protocols approved by the Institutional Animal Care and Use Committee of the University of North Carolina Chapel Hill.

Frances S. Li, along with Kathryn S. Carpentier and Thomas E. Morrison (Department of Immunology and Microbiology, University of Colorado School of Medicine, Aurora, CO, USA), discussed defining MARCO–virus interactions that are important for alphavirus clearance from the circulation. Arboviruses, such as mosquito-borne alphaviruses, are major public health concerns, and the capacity of an arbovirus to be transmitted in a human–mosquito–human transmission cycle has fueled disease outbreaks worldwide. Major determinants of arbovirus transmission, geographic spread, and pathogenesis are the magnitude and duration of viremia in the vertebrate host. Previously, they determined that multiple arthritogenic alphaviruses, including chikungunya (CHIKV) and Ross River (RRV) viruses, are cleared efficiently from murine circulation by scavenger receptor A6 (MARCO) expressed on liver macrophages. Here, they find that MARCO-dependent clearance of CHIKV is contingent on the presence of specific biochemical features of the virion surface E2 and E1 glycoproteins: a lysine (K) residue at E2-200 (K200), a negative charge at E2-208, and a positive charge at E1-61. Similar distinct electrostatic requirements were also observed for RRV clearance, where a lysine residue is required at E2-251 (K251), a basic histidine (H) residue at E2-232 (H232), and an acidic aspartic (D) residue at E2-246 (D246). Utilizing an in vitro cell culture system, they uncovered that ectopic expression of MARCO promoted internalization of CHIKV and RRV particles, and this effect was dependent on the scavenger receptor cysteine-rich (SRCR) domain of MARCO, which displayed distinct electrostatic distributions. Collectively, these findings suggest that CHIKV and RRV particles ionically interact with MARCO SRCR domain via unique charge characteristics surrounding CHIKV E2 K200 and RRV E2 K251. Ultimately, this project may provide new insight into the molecular mechanisms that dictate arthritogenic alphavirus transmission, dissemination, and pathogenesis in vertebrate hosts. This work was supported by the following funding sources: AI123348, AI148144, and AI140567. All mice experiments were performed at the University of Colorado, Anschutz Medical Campus and adhere to the Institutional Animal Care and Use Committee (IACUC) guidelines.

Cormac J. Lucas, along with Ryan M. Sheridan, Bennett J Davenport, Jay R. Hesselberth, and Thomas E. Morrison (Department of Immunology and Microbiology, University of Colorado Anschutz Medical Campus; RNA Bioscience Initiative, University of Colorado Anschutz Medical Campus, Aurora, CO, USA), discussed how Chikungunya virus interacts with floor and Marco+ LECs in the draining lymph node early after infection. Pathogenic Chikungunya virus (CHIKV) strains evade B cell responses to establish persistent infection and induce infiltration of the draining lymph node (dLN) with inflammatory iNOS-expressing myeloid cells that impair normal B cell responses and disrupt normal lymph node organization. The LN cells that interact with pathogenic CHIKV strains to initiate disruption of normal lymphoid tissue function remain to be identified. In prior studies, they found that accumulation of CHIKV in the dLN is dependent on expression of the scavenger receptor MARCO. Using single-cell RNA sequencing of murine LN non-hematopoietic cells at two early timepoints, 8 and 24 h post-infection, they found that CHIKV RNA accumulates predominantly in two subsets of lymphatic endothelial cells (LECs), floor LECs, and MARCO+ LECs, with increasing MARCO expression correlating with higher viral RNA counts. Moreover, both of these LEC subsets lack expression of Mxra8, a CHIKV cell entry receptor, suggesting that MARCO facilitates CHIKV internalization in LECs. Decreased expression of host genes, increased subgenomic viral RNA counts in CHIKV+ cells, and the disappearance of the floor LECs by 24 h suggest that floor and MARCO+ LECs may support viral replication. A subset of LN cells upregulate innate immune genes at 8 h, but the LN displays a dominant ISG response by 24 h. Further work will investigate the susceptibility and permissiveness of LECs to CHIKV infection in vitro and identify innate immune signaling pathways regulated by MARCO in LECs to better understand the contribution of these cells to immune responses initiated in the dLN. All animal studies were performed following guidelines and protocols approved by the Institutional Animal Care and Use Committee of The University of Colorado Anschutz Medical Campus.

Gabriela Ramirez ^1^, along with Paul S. Soma ^1^, Nunya Chotiwan ^1^, Nurul Islam ^1^, Barbara Graham ^1^, Austin J. Mejia ^2^, Laura St Clair ^1^, John T. Belisle ^1^, Elizabeth A. McGraw ^2^, and Rushika Perera ^1^ (^1^ Department of Microbiology, Immunology and Pathology, Colorado State University, Fort Collins, CO; ^2^ Biology Department and The Center for Infectious Disease Dynamics, Pennsylvania State University, University Park, PA 16802), discussed her work investigating the metabolic differences that define virus–host interactions between arboviruses and *Aedes aegypti* mosquitoes. The metabolic landscape, also referred to as the metabolome, plays a critical role in the replication of arboviruses in *Ae. aegypti* mosquitoes. As obligate parasites, viruses must strike a balance between commensalism and competition within their hosts for effective viral entry, replication, and transmission. Insecticides, ecological conditions, geographic distribution, age of vector, and endosymboints (such as *Wolbachia*) also alter metabolic conditions in mosquitoes and influence vector competence. To investigate which host resources are required and/or altered by arbovirus infection, they used high-resolution mass-spectrometry-based metabolomics approaches combined with loss-of-function analyses in *Ae. Aegypti* infected with Zika (ZIKV), dengue (DENV), and Chikungunya (CHIKV) viruses. Intriguingly, they found that multiple lipid classes are altered. Major components of cellular membranes, such as phosphoglycerolipids, were increased in parallel with active viral replication in the midgut. Certain sphingolipids were also altered at early times post-infection. These are particularly interesting, as they are well-characterized as potent bioactive signaling molecules in mammals. However, their role in mosquito biology remains unknown. Additionally, they were able to identify unique features that metabolically distinguish ZIKV, DENV, and CHIKV infection with the *Ae. aegypti* vector. These data lend insight into how unique metabolic biosignatures induced by these three viruses can inform on tolerance of co-infection. Progress in these studies was presented. No animal or human studies were performed.

Molly Ring, along with Anna-Sophia Leon, Ashley Janich, Paula Lado, Nguyen C., Pugh G., and Brian Foy (Center for Vector-Borne Infectious Diseases, Department of Microbiology, Immunology, and Pathology, Colorado State University, Fort Collins, CO, USA), presented her work on the efficacy of ivermectin and convectional insecticides against wild-type West Nile virus vectors from urban and rural Larimer County. Colorado has some of the highest West Nile virus (WNV) case numbers in the United States each year, and perennially has counties with some of the highest WNV disease incidence. Current WNV control is limited to applying mosquito larvicides to water sources and spraying insecticides to control adult mosquito vectors in residential areas when risk of WNV transmission is high. The latter has limited proof of efficacy, can be poorly targeted, has environmental toxicity concerns, and may be ineffective against mosquito populations if they are already resistant to similar insecticides applied to residential and agricultural areas. Wild birds are WNV reservoirs because they infect local mosquito vectors that feed upon them, and these vectors subsequently transmit WNV to humans. They are developing an alternative WNV control strategy that treats birds with the drug ivermectin (IVM) to kill mosquitoes that blood feed upon them to reduce WNV transmission risk to humans. They investigated the susceptibility of local rural and urban wild-type *Culex tarsalis* to the adulticide permethrin using insecticide bioassays and to IVM by blood feeding them on IVM-treated chickens and observing their survivorship in semi-field mesocosms they constructed. Bioassays determined that the lab strain was susceptible to permethrin, and the rural strain was deemed possibly resistant, but future testing is needed to confirm this. There was significantly lower survivorship in mosquitoes that fed on IVM-treated birds compared to the control group. These preliminary data suggest that they can potentially implement this control method in the field to help reduce WNV transmission. Funding for this project was provided by NIH grant R01AI148633. All animal studies were performed following guidelines and protocols approved by the Institutional Animal Care and Use Committee of Colorado State University.

Paul S. Soma ^1^, along with Rebekah C. Gullberg ^1^, Barbara G. Andre ^1^, Kimberly Anderson ^1^, Stephanie Mills ^1^, Elena Lian ^1^, Kristen Krieger ^1^, Lionel Gresh ^2^, Raquel Burger-Calderon ^5^, Amber Hopf-Jannasch ^3^, Angel Balmaseda ^4^, Barry Beaty ^1^, Eva Harris ^5^, Carol D. Blair, ^1^ and Rushika Perera ^1^ (^1^ Dept. of Microbiology, Immunology and Pathology, Colorado State University, Fort Collins, CO, USA; ^2^ Sustainable Sciences Institute, Managua, Nicaragua; ^3^ Metabolite Profiling Facility, Bindley Bioscience Center, Purdue University, West Lafayette, IN, USA; ^4^ Laboratorio Nacional de Virología, Centro Nacional de Diagnóstico y Referencia, Ministry of Health, Managua, Nicaragua; ^5^ Division of Infectious Diseases and Vaccinology, School of Public Health, University of California, Berkeley, CA, USA), shared their research on the identification of metabolic biomarkers that predict dengue disease severity. Dengue viruses (DENVs) place over 2 billion people at risk of infection each year, rendering them the most aggressive arboviruses worldwide. There are four serotypes of DENVs. Infection with one serotype does not cross-protect from infection with other serotypes. No treatment options exist due to complications in disease pathology mediated by the immune response. Using a liquid chromatography–high-resolution mass spectrometry (LC-MS) untargeted metabolomics approach, they identified a small metabolite biosignature panel in acute-phase pediatric patient serum that is associated with dengue disease severity and pathogenesis. This metabolite panel has high potential for early prediction of dengue disease severity and improved clinical management. Additionally, metabolite biosignatures for Zika virus and Chikungunya infections have been established. Untargeted LC-MS analysis provides putative identification of detected metabolites through chromatographic retention time (RT) and accurate mass (*m/z*) measurements of metabolite ions (e.g., [Metabolite+H]^+^). Putatively identified metabolites must be further validated using tandem mass spectrometry (MS/MS) methods and/or RT matching with synthetic standards. Collision-induced dissociation (CID) of chromatographically resolved serum metabolites (LC-MS/MS analysis) was performed in a ‘TopN’ data-dependent manner with an inclusion list for metabolites of particular interest. CID of metabolite parent ions generated product ions that provided metabolite structural information. Experimental CID spectra for putatively identified metabolites were searched against MS/MS spectral databases (mzCloud, METLIN, HMDB) or compared to CID spectra from synthetic standards when available. Confident identification of metabolite biomarkers of dengue, Zika, and Chikungunya diseases aids in elucidating relevant metabolomic pathways and lays a foundation for simplified point-of-care prognosis. All human studies were performed following guidelines and protocols approved by the Institutional Review Board of Colorado State University.

Laura St. Clair ^1^, along with Carissa Drake ^2^, Michael Spedding ^3^, David Priestman ^2^, Frances Platt ^2^, and Rushika Perera ^1^ (^1^ Center for Vector-borne Infectious Diseases, Department of Microbiology, Immunology and Pathology, Colorado State University, Fort Collins, CO, USA; ^2^ Department of Pharmocology, University of Oxford, Oxford, UK; ^3^ Spedding Research Solutions SAS, Le Vesinet, France), presented on her work analyzing the effect of inhibition of glucosylceramide hydrolysis during DENV2 infection in human hepatoma cells. Sphingolipids (SLs) are potent bioactive signaling molecules involved in nearly all major biological responses. Previously, her colleagues and others showed that there are significant alterations of SL metabolism during infection with dengue viruses (DENVs) in both human and mosquito hosts. Her colleagues demonstrated that disruption of de novo ceramide biosynthesis in the mosquito reduces DENV2 genome replication and infectious virus release. They also demonstrated that there are significant differences in the SLs found in human sera between the febrile, defervescent, and convalescent phases of dengue disease. Others have shown that dysregulation of SLs is evident in vascular leakage and thrombocytopenia. However, it remains unclear how viral gene products might alter SLs during infection or what role specific SLs play in the viral lifecycle. In an siRNA-mediated screen of enzymes within the SL pathway, she discovered that losses of function of the two enzymes responsible for hydrolysis of glucosylceramide (GluCer) have opposite effects on DENV2 replication and release. Using chemical modulators, she confirmed that both the inhibition of non-lysosomal beta-glucocerebrosidase (GBA2) and upregulation of the lysosomal GBA1 enzyme reduce viral replication and release. These data suggest that subcellular location of GluCer hydrolysis is critical for the DENV2 lifecycle. She systematically investigated how specific parts of the DENV lifecycle are affected by these enzymes. Additionally, she completed analyses to determine their activity over time and determined how they are modulated during infection. Here, she presented an update on the progress of these studies. No animal or human studies were performed.

Elisa Thrasher, along with Emma Harris and Rebekah Kading (Department of Microbiology, Immunology, and Pathology, Colorado State University), investigates the dynamic bacterial community of *Aedes aegypti.* Arbovirus (arthropod-borne virus) transmission by *Aedes aegypti* mosquitoes is a worldwide public health crisis that requires in-depth research for novel methods to disrupt viral transmission. The mosquito midgut harbors a multitude of bacterial endosymbionts that directly interact with invading viruses entering through an infectious blood meal. Various bacterial species have been identified as constitutive members that are naturally present in field-collected and lab-reared *Ae*. *aegypti* microbiota and have been shown to provide immunity to entomopathogens and vulnerability to arboviruses, bacterial translocation, and antibiotic resistance. Understanding the microbial composition and its relationship to the mosquito is an essential step in developing efficient paratransgenic techniques that disrupt viral transmission. This study investigated the composition and plasticity of the microbiota of adult female *Aedes aegypti* mosquitoes. They collected female *Ae*. *aegypti* mosquitoes (n = 8–10) and surface sterilized, lysed, and plated them onto non-selective agar using time-course sampling methods (at emergence, 1, 2, and 3 weeks post-emergence). A total of 104 of the 232 nonselective agar plates developed observable bacterial colonies, from which DNA was extracted. Unique phenotypic colonies were propagated at a biologically relevant temperature of 28 °C and identified via Sanger sequencing of the 16s ribosomal RNA gene. Future works to examine shifts in bacterial composition through application of antibiotics as a means of selective pressure would, when taken together with these data, provide a larger, novel snapshot of the various bacterial species within the midgut that could be targeted for genetic manipulation.

Brian C. Ware, together with Bennett J. Davenport and Thomas E. Morrison (Department of Immunology and Microbiology, University of Colorado School of Medicine, Aurora, CO, USA), presented work on how CHIKV-infected cells display reduced MHC Class I expression and a reduced capacity to activate CD8 T cells. Arthritogenic alphaviruses, including Chikungunya (CHIKV), Mayaro (MAYV), and Ross River (RRV) viruses, are re-emerging global health threats with no approved vaccines or therapies. Infection with these viruses causes debilitating pain and inflammation in musculoskeletal tissue for months to years due partially to the persistence of viral RNA and antigens. Prior studies revealed that arthritogenic alphavirus infection evades the B cell response to establish persistence. However, mechanisms by which these infections evade CD8 T cell responses, another critical arm of antiviral adaptive immunity, are undefined. Indeed, anti-CHIKV CD8 T cells are present in lymphoid tissues and sites of infection, and these cells can kill peptide-loaded target cells. Remarkably, genetic deletion of CD8 T cells does not alter CHIKV burdens in joint-associated tissues during acute or chronic infection, leading them to hypothesize that infected cells escape surveillance by recruited CD8 T cells. To investigate this idea, CHIKV-infected joint cells, including fibroblasts and myeloid cells, were assessed for MHC-I cell surface expression. They found marked loss of MHC-I expression coincident with high levels of viral replication. Furthermore, in vitro infected fibroblasts failed to activate CD8 T cells above the bystander background, suggesting that CHIKV infection suppresses the antigen presentation capacity of infected cells. Lastly, mutating the nonstructural protein 2 of CHIKV ameliorates the reduction in MHC-I expression, suggesting a role for nsP2 in the evasion of the CD8 T cell response and establishment of persistent infection. These results highlight a role for CHIKV nsP2 in evading the CD8 T cell response and contributing to CHIKV persistence. No animal or human studies were performed.

Joseph Westrich (Department of Microbiology, Immunology, and Pathology, Colorado State University) presented about the longitudinal viral progression and immunological responses to Bluetongue Virus 17 in experimentally infected sheep. Bluetongue virus (BTV) is an economically important arthropod-borne pathogen that infects ruminant species worldwide. The severity of BTV infections range from asymptomatic to lethal, with the most severe cases succumbing to disease within one week. Animals that survive the infection often require months to fully recover. The immune response to BTV infection is thought to contribute to the propagation of disease in addition to being critical in the ultimate resolution of infection. Although BTV has been recognized since the 1800s, much of the cellular and cytokine response remains poorly understood due to limited reagent availability for the natural host species. To gain a greater understanding of the role the immune response plays in BTV infection, he infected a cohort of sheep with BTV-17 and longitudinally monitored each for clinical disease, viremia, and specific immunological parameters. Immune cells and cytokines were evaluated with traditional flow cytometry, RNA flow cytometry, RT-qPCR, and/or fluorescent-based antibody arrays. All BTV-inoculated sheep exhibited clinical signs characteristic of BTV disease. Circulating virus was observed as early as 3 days post=inoculation (dpi) and remained detectable for the remainder of the study (24 dpi). A distinct pan-leukopenia was observed between 8 and 14 dpi that rebounded to mock-inoculated control levels at 17 dpi. He observed increased expression of pro-inflammatory cytokines after 8 dpi—notably, the pro-inflammatory cytokine CXCL10. Taken together, he established a sheep model of BTV infection and successfully monitored the longitudinal immunological response and viral progression using a combination of traditional methods and cutting-edge technology.

### 2.4. Viral Detection and Other Novel Techniques

Noelia Altina ^1,^ along with David Maranon ^1^, John Anderson ^1^, and Jeff Wilusz ^1^ (^1^ Microbiology, Immunology, Pathology Department, Colorado State University, Fort Collins, CO, USA), investigated fundamental knowledge gaps in the RNA biology of SARS-CoV-2. Specifically, they are interested in the molecular mechanisms underlying SARS-CoV-2 mRNA capping, cap-proximal adenosine methylation, and the impact of viral RNA–protein interactions on host cell RNA biology. First, to characterize the putative role of the viral Nsp12 protein as the guanylyltransferase involved in SARS-CoV-2 mRNA capping, they successfully expressed and purified recombinant protein. They are currently performing biochemical assays and mutagenesis studies to gain insight into enzymatic mechanisms and potential drug targets. Second, they obtained evidence suggesting that the A residue at the 5′ end of SARS-CoV-2 mRNAs likely contains an ‘m6Am’ methylation modification. This is significant because all cellular mRNAs that initiate at an A residue contain a similar m6Am modification. Thus, they hypothesize that SARS-CoV-2 is modifying its mRNAs to prevent their detection as ‘non-self’. They are currently testing this hypothesis and will identify the mechanism of SARS-CoV-2 m6Am RNA modification. Finally, two cellular proteins (hnRNPA1 and PTBP) that regulate alternative splicing bind to the abundant viral mRNAs in related coronaviruses and become mis-localized to the cytoplasm during infection. Therefore, they hypothesize that SARS-CoV-2 mRNAs can sequester these splicing factors, leading to significant changes in alternative mRNA splicing. RT-PCR analyses of the splicing patterns across select exons of hnRNPA1/PTBP-targeted pre-mRNAs confirm this hypothesis and suggest a new mechanism for the impact of SARS-CoV-2 infection on cellular RNA biology. These studies collectively focus on attractive targets for developing novel broad-spectrum anti-coronavirus drugs. No animal or human studies were performed.

Ali L. Brehm, along with Mark Stenglein (Department of Microbiology, Immunology, and Pathology, Colorado State University, Fort Collins, CO, USA), presented their work on creating a homozygous fly line utilizing non-lethal genotyping. *Drosophila melanogaster* is used widely as a model organism due to its high number of orthologous genes with humans. New lines of flies are created regularly that contain and express genes of interest through multiple generations of inbreeding. Inbreeding can lead to the maintenance of deleterious mutations and creates stocks of *D. melanogaster* that are considered weak, and which may be more susceptible to pathogens that create mass die-offs. Using non-lethal DNA extraction and PCR, a fly line that was homozygous for an endogenous viral element (EVE) that was 95% similar to the RNA-dependent RNA polymerase gene of galbut virus was created. Galbut virus is a double-stranded RNA multipartite partitivirus that is of particular interest because it is found in 100% of wild populations of *D. melanogaster* and has the potential to be used in cross-species biocontrol efforts, but despite being found in all populations, only 60% of individuals are infected. More information regarding *D. melanogaster* resistance to galbut virus is needed to determine if galbut virus EVEs may limit its use in biocontrol. Non-lethal genotyping has the potential to allow the revival of significant fly lines that are weak due to inbreeding depression, as was the case in the original galbut EVE fly line. Funding for this project was provided by NSF IOS 2048214. No animal or human studies were performed.

Luke Davis, along with Philip Fox, Kaitlyn Wagner, Liddy Gordon, and Mark Zabel (Department of Microbiology, Immunology, and Pathology, Colorado State University, Fort Collins, CO, USA), presented an update on CSU’s efforts to develop a high-throughput pooling and ddPCR-based strategy for COVID-19 screening of asymptomatic populations. Screening of asymptomatic populations for the presence of SARS-CoV-2 is an effective way to permit in-person classes and gatherings while still ensuring the health and safety of those involved. However, the costs of periodic screening and the materials and reagents required can be prohibitive of such a strategy, especially during a pandemic, when common reagents become difficult to obtain. They developed a paired-pooling strategy that was implemented for a droplet digital PCR assay to detect SARS-CoV-2 in saliva, which reduced costs (<10 USD/sample) and reagent consumption while supporting high-throughput (>2000 samples/day). This assay was implemented in an asymptomatic screening program at CSU, and in the Fall 2020 and Spring 2021 semesters, over 150,000 samples were screened. The centerpiece of this strategy is the paired-pooling approach, in which each sample is added to a set of two pools such that no two pools contain more than one common sample. With this approach, one positive sample can be identified out of 64 total samples without requiring a rerun of all the samples within the positive pools. As the percentage of positive samples increases, the number of required reruns grows with the square of the number of positive samples, so this approach works best for screening asymptomatic populations with a low percentage of positive samples. The paired-pooling approach can be implemented with different assays and specimen types and represents an effective way to perform asymptomatic screening while keeping costs and material use low. All human studies were performed following guidelines and protocols approved by the Institutional Review Board of Colorado State University.

Nicole Ehrhart ^1,2^, along with Greg Ebel ^2^ and Kristy Pabilonia ^2^ (^1^ Columbine Health Systems Center for Healthy Aging and Department of Clinical Sciences, ^2^ Department of Microbiology, Immunology, and Pathology, Colorado State University, Fort Collins, CO, USA), presented her group’s surveillance efforts of staff at nursing homes and assisted living facilities. Nursing homes and assisted living facilities, collectively known as long-term care facilities (LTCFs), provide care for some of the most vulnerable populations in society. Communal living spaces and the need for daily contact between staff and residents creates an opportunistic environment for the spread of any infectious disease. In particular, the morbidity and mortality within LTCFs throughout the COVID-19 pandemic has demonstrated the extreme vulnerability of both LTCF residents and staff to transmissible viral illness. To detect pre- and asymptomatic infections among LTCF staff, they performed weekly SARS-CoV-2 surveillance testing of staff and residents at 31 LTCFs over 30–60 consecutive weeks. They collected infection and outbreak patterns, genomic sequencing data, serum neutralizing antibody data, and the success or failure of various infection mitigation strategies. They archived one of the largest known collections of longitudinal diagnostic specimens. They concluded that: (1) LTCF staff represent a disproportionally high percentage of SARS-CoV-2 infections as compared with non-healthcare community members regardless of whether or not LTCF staff have direct patient contact, (2) LTCF staff who tested positive were most likely to be infected from exposure within their workplace and not the community, (3) early identification of infected staff via regular surveillance testing was effective at mitigating outbreaks, and (4) the testing cadence and type was highly correlated with mitigation efficacy. Infectious disease response agility and future pandemic preparedness will require more proactive strategies to prevent the high morbidity and mortality within LTFCs in the future.

Emmanuel Ijezie and Elizabeth Fortunato (Department of Biological Sciences University of Idaho, Moscow, ID, USA) presented their research investigating the role that Nidogen-1 plays in aberrant neurodevelopment of HCMV-infected brain organoids. The ability to generate three-dimensional brain organoids in vitro has provided an ethical system to model and study viral–host interactions within a developing human brain. Congenital human cytomegalovirus (HCMV) is the leading cause of viral-induced birth defects in humans. These birth defects include microcephaly, sensorineural hearing loss, and cognitive impairment. The process in which the developing fetus incurs these neurological defects is still poorly understood. To elucidate some of these mechanisms, their lab has adapted a brain organoid development protocol that utilizes HCMV-infected induced pluripotent stem cells to generate in vitro brain organoid structures that model the first and second trimester in fetal brain development. Early in development, brain organoids generate neural rosette structures that model neural tube formation in vertebrates. These neural rosettes further develop into structures with multiple neural cell layers. Both infected and uninfected brain organoids were collected, frozen, and sectioned for qualitative and quantitative histological and immunofluorescence (IF) protein analysis. Histological analysis of infected brain organoids revealed that fewer neural rosettes were formed. IF analysis of neural rosettes showed that nidogen-1 protein expression in the basal lamina was greatly reduced in infected neural rosettes compared to uninfected neural rosettes. IF analysis also indicated that expression of neural progenitor cell protein markers Pax-6 and Nestin was downregulated in infected neural rosettes compared to uninfected neural rosettes. IF analysis of dissociated brain organoids detected viral proteins IE1 and PP71 early during organoid development. Infected cortical structures also displayed decreased B-tubulin III expression, indicating a delay in neurogenesis. In conclusion, the progression of neural development is disrupted in neural rosettes within infected brain organoids. This disruption in the development and downregulation of cellular proteins correlates with the reanimation of viral proteins within these brain organoids. Understanding the role of HCMV in downregulating neural developmental processes in brain organoids could elucidate the mechanisms behind the disease phenotypes seen in congenitally infected newborns. This study was funded by NIH RO1 AI051463 and AI139503 to E.A.F., NIH P20 RR016454. No animal or human studies were performed.

Tanya Jolly ^1^, along with Luke Davis ^1^, August Luc ^1^, Jim Huang ^1^, Susan De Long ^2^, Philip Fox ^1^, and Carol Wilusz ^1^ (^1^ Wastewater Testing Laboratory/Molecular Quantification Core, Dept. of Microbiology, Immunology, and Pathology, and ^2^ Department of Civil & Environmental Engineering, Colorado State University), discussed a robust and reproducible assay for monitoring SARS-CoV2 in wastewater from treatment plants and college dormitories. Wastewater-based epidemiology represents an unbiased, cost-effective, and non-invasive means of monitoring a population for infectious agents. SARS-CoV2 infects the human gastrointestinal tract and is shed in feces prior to establishing itself in the respiratory system, from which it can be disseminated to others and causes most of its symptoms. This means that circulating virus can often be detected in the sewer prior to cases arriving at the clinic. They developed a molecular assay to detect SARS-CoV2 in wastewater that is sensitive, rapid, and resistant to inhibition. Since August 2020, their facility has received up to 100 samples a week of wastewater from university dorms and wastewater treatment plants across the Colorado Front Range. Their protocol allows concentration of viral particles via ultrafiltration, extraction of viral RNA, and quantification of the SARS-CoV2 genome copy number using probe-based multiplex droplet digital PCR. Data are reported to stakeholders within 24 h of sample receipt and inform public health messaging and decision making. Variant strains can be detected by further analysis through sequencing. Acknowledgements: Their assay was developed in collaboration with GT Molecular Inc., the Colorado Department of Public Health and Environment, the Colorado Wastewater Collaborative with funding from the Colorado State University Office of the Vice President for Research, and Metro Water Recovery.

Lexi Keene, along with Tillie Dunham and Mark Stenglein (Department of Microbiology, Immunology, and Pathology, Colorado State University, Fort Collins, CO, USA), discussed her testing efforts involved in building a better fly trap for catching wild flies. *Drosophila melanogaster* have been utilized since the early 1900s, and these insects have contributed substantially to the understanding of the natural world. Most *Drosophila* research has relied on lab-reared strains that are inbred and long removed from their geno-/phenotypes. Studying wild flies offers a way to extend the relevance of this laboratory model. Her PhD research is focused on studying viruses that naturally infect *D. melanogaster*, which can reveal principles of host–virus interactions that can further the study of disease in animals and plants. To accomplish this, she needed a method to efficiently trap live flies. Traps baited with banana and yeast have been an effective way to capture wild flies; however, the bait decomposes rapidly, making it difficult for flies to lay eggs and for adults to be retrieved. Thus, the ability to capture wild flies needs to be through the use of food that is stable over time and during transportation while also providing a place for the flies to reproduce. Three types of food were tested for longevity in different environments. Cornmeal food proved the most stable. Additionally, four additives were tested to determine which would attract flies. The flies were overwhelmingly attracted to food that had a banana component and, surprisingly, were almost repulsed by the addition of ethyl isovalerate, which had been identified as the key attractant in marula fruit, the purported ancestral food source of *D. melanogaster*. She is continuing to optimize the bait composition and the efficacy of these traps in real-world environments. The work was supported by NSF grant #2048214 and qCMB T32: T32GM132057. All animal studies were performed following guidelines and protocols approved by the Institutional Animal Care and Use Committee of Colorado State University.

Stefan L. Oliver (Department of Pediatrics—Infectious Diseases, Stanford University School of Medicine, California, USA) presented his lab’s work studying the cell-fusion-dependent pathogenesis of varicella-zoster virus (VZV). VZV is an alphaherpesvirus of medical importance and is the causative agent of chicken pox (varicella) and shingles (zoster). Although considered benign diseases, both varicella and zoster can produce complications. Zoster is painful, potentially leading to protracted post-herpetic neuralgia. VZV has also been linked to stroke and has been related to giant cell arteritis in some cases. A hallmark of VZV pathology is the formation of multinucleated cells driven by cell–cell fusion (abbreviated as cell fusion) mediated by the VZV glycoproteins gB, gH, and gL, which constitute the fusion complex of herpesviruses, including VZV. These evolutionarily conserved glycoproteins are required for virion entry into cells, with the gH-gL heterodimer priming the trimeric gB fusogen to complete the membrane fusion process. The adaptive immune response can target the fusion complex, producing antibodies that bind to both the gB trimer and the gH-gL heterodimer. An Achilles’ heel for VZV and a new functional domain for gB were revealed by cryo-EM and X-ray crystallography derived near atomic resolution structures of neutralizing antibodies bound to these critical glycoproteins. Expression of gB, gH, and gL during VZV infection and trafficking to the cell surface enables cell fusion, which is inhibited by these neutralizing antibodies. Moreover, recent evidence supports the concept that cellular processes are required for regulating cell fusion induced by gB/gH-gL. Mutations within the carboxyl domains of either gB or gH have profound effects on fusion regulation and dramatically restrict the ability of VZV to replicate in human skin. This loss of regulation modifies the transcriptome of VZV-infected cells. Furthermore, cellular proteins have significant effects on the regulation of gB/gH-gL-mediated cell fusion and the replication of VZV, exemplified by the cellular phosphatase calcineurin. His presentation focused on the current state-of-the-art knowledge about the molecular controls of cell-fusion-dependent pathogenesis caused by VZV. This research was supported by a Stanford Bio-X Interdisciplinary Initiatives Seed Grant and the National Institutes of Health through grants P41-GM103832, R01-GM079429, R01-AI102546, R37-AI20459, and S10-OD021600. All animal studies were performed following guidelines and protocols approved by the Institutional Animal Care and Use Committee of Stanford University.

Carol Wilusz ^1,^ along with Phil Fox ^1^, Laura Bankers ^2^, Shannon R. Matzinger ^2^, Brian Erly ^2^, Joshua Goldmann-Torres ^3^, Mazdak Arabi ^4^, and Susan De Long ^4^ (^1^ Department of Microbiology, Immunology, and Pathology, Colorado State University, ^2^ Colorado Department of Public Health and Environment, ^3^ Metro Water Recovery, ^4^ Department of Civil and Environmental Engineering, Colorado State University, Fort Collins, CO, USA), presented on their efforts to establish wastewater-based epidemiology (WBE) at Colorado State University. WBE has been established as a viable, valuable, and cost-effective means of monitoring infectious disease within a community. SARS-CoV-2 is shed in feces as early as one to two days post-infection, and viral RNA is detectable for several days in wastewater. Thus, detection and quantification of its genome can be used to evaluate trends in infection rate and inform public health actions. Over the last year, they have monitored wastewater from 21 treatment plants across the state of Colorado for SARS-CoV-2 and correlated the data to the clinical caseload in the population they represent through census tracts. The viral RNA load over time closely correlates with and slightly precedes increases in reported clinical cases. The ability to track the virus through wastewater is more valuable than ever as the epidemiology of SARS-CoV-2 changes with increased vaccination, new variants, and changing testing and infection patterns. Moreover, the isolated RNA can be used to detect the presence of novel and known variants of the virus. For example, mutations associated with the Alpha and Delta variants have been detected via mutation-specific PCR and/or whole-genome sequencing prior to clinical cases being observed within the Colorado population.

### 2.5. Antivirals, Treatments, and Vaccines

Corey L. Campbell ^1,^*, along with Trey K. Snell ^1^, Susi Bennett ^1^, John Wyckoff ^2^, Darragh Heaslip ^2^, Emma K. Harris ^1^, Daniel Hartman ^1^, Elena Lian ^1^, Brian H. Bird ^3^, Mark D. Stenglein ^1^, Richard A. Bowen ^1^, and Rebekah C. Kading ^1^ (**^1^** Arthropod-borne & Infectious Diseases Laboratory, Department of Microbiology, Immunology, and Pathology, Colorado State University, Fort Collins, CO, USA. ^2^ Department of Neurology, University of Colorado School of Medicine, Boulder, CO, USA. ^3^ College of Veterinary Medicine and Biomedical Sciences, Colorado State University, Fort Collins, CO, USA), presented her work on human vaccine candidate development against Rift Valley fever virus (RVFV). RVFV is a mosquito-borne pathogen with significant human and veterinary health consequences that periodically emerges in epizootics. RVF causes fetal loss and death in ruminants, and in humans, it can lead to liver and renal disease, delayed-onset encephalitis, retinitis, and, in some cases, severe hemorrhagic fever. A live attenuated vaccine candidate, DDVax, was developed by the deletion of the virulence factors NSs and NSm from a clinical isolate, ZH501, and has proven safe and immunogenic in rodents, pregnant sheep, and non-human primates. Deletion of NSm also severely restricted mosquito midgut infection and inhibited vector-borne transmission. To demonstrate environmental safety, this study investigated the replication, dissemination, and transmission efficiency of DDVax in mosquitoes following oral exposure compared to RVFV strains MP-12 and ZH501. Infection and dissemination profiles were also measured in mosquitoes 7 days after feeding on goats inoculated with DDvax or MP-12. Hypothesis: DDVax should infect mosquitoes at significantly lower rates than other RVF strains and, due to lack of NSm, be transmission incompetent. Exposure of *Ae. aegypti* and *Cx. tarsalis* to 6–8 log10 plaque-forming units (PFU)/mL DDVax by artificial bloodmeal resulted in significantly reduced DDVax infection rates in mosquito bodies compared to controls. Plaque assays indicated negligible transmission of infectious DDVax in *Cx. tarsalis* saliva (1/140 sampled) and none in *Ae aegypti* saliva (0/120). Serum from goats inoculated with DDVax or MP-12 did not harbor detectable infectious virus according to the plaque assay at 1, 2, or 3 days post-inoculation; infectious virus was, however, recovered from mosquito bodies that fed on goats vaccinated with MP-12 (13.8% and 4.6%, respectively), but strikingly, DDvax-positive mosquito bodies were greatly reduced (4% and 0%, respectively). Furthermore, DDVax, unlike MP-12, did not disseminate to legs/wings in any of the goat-fed mosquitoes. Collectively, these results are consistent with a beneficial environmental safety profile. All animal studies were performed following guidelines and protocols approved by the Institutional Animal Care and Use Committee of Colorado State University.

Chaoping Chen ^1^, along with Liangqun Huang ^1^ and Megan Gish ^1^ (^1^ Biochemistry and Molecular Biology Department, Colorado State University, Fort Collins, CO, USA), presented on a main protease (M^PRO^) of SARS-CoV-2 that is indispensable for viral replication. It is initially synthesized as part of polyprotein precursors whose autoproteolysis liberates free mature M^pro^. They herein studied autoprocessing of fusion precursors with the mature M^pro^, along with a few flanking amino acids (to keep the cleavage sites) sandwiched between tags in transfected mammalian cells. Mutating the conserved Gln of C-terminal P1 residue to Glu abolished C-terminal autoprocessing, while it had no impact on N-terminal autoprocessing. Interestingly, mutating the conserved N-terminal P1 Gln to Glu did not ablate N-terminal processing, indicating distinct catalysis kinetics at N- and C-terminal autoproteolysis. Various upstream fusion tags (sGST, GST, CD63, Nsp4) also showed diverse N-terminal autoprocessing efficiencies, suggesting regulation of M^pro^ precursor catalysis in the polyprotein context. Furthermore, mutation QtoE at the N-terminal P1 position altered precursor catalysis and outcomes, as demonstrated by different susceptibilities to several preclinical M^pro^ inhibitors. N-terminal processing of precursors with Gln at P1 was more resistant to drug inhibition than those with Glu at P1 in the GST-fusion context. Additionally, the mature M^pro^ released from Q precursors was self-degradation prone and could be suppressed by boceprevir, calpain inhibitor II, and GC376, whereas those released from E precursors were self-degradation resistant and irresponsive to drug treatment. Many amino acid variations at the N-terminal P1 position were well tolerated with various processing efficiencies, but a few containing β-branched side chains blocked N-terminal processing. These results collectively revealed substrate diversity and catalysis plasticity of fusion precursors, providing insights into drug discovery targeting the M^pro^. No animal or human studies were performed.

Rupika Delgoda ^1^, along with S. Elmegerhi ^2^, W. Irvine ^1^, D. Picking ^1^, S. Francis ^1^, and Rushika Perera ^2^ (^1^ Natural Products Institute, University of the West Indies, Mona, Jamaica; ^2^ Center for Vector-Borne Infectious Diseases, Colorado State University, Fort Collins, CO, USA), presented their work on evaluating the potential of Jamaican ethnomedicines for their efficacy against infectious diseases. Targeting novel chemical scaffoldings entrapped in nature’s resources has yielded valuable pharmaceuticals for current medical use. Despite modern combinatorial capacities, bioprospecting of microbial, terrestrial, and marine organisms remains a thriving endeavor, due largely to their unique molecular offerings. Probing biodiversity with a history of human use has also had rewarding results, often curtailing the protracted discovery pathway. Given the high cultural reliance on ethnomedicines in the Caribbean, they delved into popular practices in Jamaica in search of bioactive molecules against infectious diseases. Community-based surveys revealed that 73% of persons rely on medicinal plants for primary care, with a large portion (78%) using them to treat respiratory tract ailments. Of the 51 plant families in use, Fabaceae, Lamiaceae, Asteraceae, Malvaceae, and Piperaceae are the most commonly used, hosting multitudes of natural bioactive alkaloid, polyphenol, terpene, and flavonoid products. They highlighted plants prepared following ethnomedical extraction protocols, tested against the SARS- COV-2, and using cytoprotection and plaque assays on Vero cells. Several plant extracts showed promising results in reducing cell death due to virus infection and reducing infectious virus release. Taken together with knowledge on anti-inflammatory activity, as well as the impact on drug-metabolizing cytochrome P450 enzymes for combination therapy, a useful appraisal has emerged for these ethnomedicines as infectious diseases therapeutics. No animal or human studies were performed.

Suad Elmegerhi ^1,^*, Elena Lian ^1^, Gabriela Ramirez ^1^, Carley McAlister ^1^, Laura St. Clair ^1^, Camryn S Guenther ^1^, Rupika Delgoda ^2^, and Rushika Perera ^1^ (^1^ Department of Microbiology, Immunology, and Pathology, Colorado State University, Fort Collins, CO; ^2^ Natural Products Institute, University of the West Indies, Mona, Jamaica) discussed their lab’s efforts in developing platforms to test antivirals against SARS-CoV-2. Despite the great efforts by the international community to develop effective vaccine candidates to mitigate the COVID-19 outbreak, many new variants and new cases are still on the rise. Therefore, there is still a need for effective and readily available antiviral therapies to help reduce disease severity and escalating cases. Re-purposing FDA-approved therapeutics or compounds in the preliminary stages of approval paves a quicker path to medical treatment and is currently a focus for antiviral development. Performing more targeted testing of compounds that interfere with the general coronavirus life cycle is another route being pursued. Some compounds that include natural products have proven antiviral properties against many viruses and have been used to treat rhinovirus and influenza virus infections. In this study, they tested the antiviral activity of different drugs and investigational compounds, natural products, peptides, enzyme inhibitors, and disinfectant-treated textile materials against SARS-CoV-2. The platforms developed for antiviral testing and results were presented. No animal or human studies were performed. 

Camryn S. Guenther, along with Elena Lian, Laura St. Clair, and Rushika Perera (Center for Vector-Borne Infectious Diseases, Department of Microbiology, Immunology and Pathology, Colorado State University, Fort Collins, CO, USA), discussed her investigations into the roles of phospholipase A2 and arachidonic acid during DENV2 infection. Dengue viruses are transmitted by *Aedes aegypti* mosquitoes and cause over 400 million infections annually. These viruses hijack host lipid pathways in the cell to support their replication. A pathway of interest is arachidonic acid (AA) metabolism. AA and lysophospholipids are released by cytosolic and secreted phospholipase A2 (PLA2) enzymes from cell membrane phospholipids. This process is called the Lands cycle. AA is also a well-known precursor of antiviral inflammatory mediators. Previous studies by their laboratory have already shown that reduction of AA through a knockdown of AA-producing PLA2 isoforms (2A, 4A, and 4C) decreased infectious viral titer during a DENV2 infection. The goal of the current study was to identify why AA plays such an important role during the viral life cycle. First, they have investigated which steps of the viral lifecycle are impacted by the reduction of AA through an siRNA-mediated knockdown of PLA2 (2A, 4A, and 4C). Second, they conducted experiments to determine if exogeneous AA addition could recover infectious viral titer in the siRNA-treated cells. It is their hypothesis that PLA2 and AA may have dual roles during virus infection, both in supporting viral replication and creating the antiviral inflammatory precursor molecules. Progress on this work was presented. No animal or human studies were performed. 

Jim Heath (Institute for Systems Biology, Seattle, WA, USA) presented on research from his lab about quantitative analysis of post-acute sequelae in COVID-19 patients. Post-acute sequelae from COVID-19 (PASC) represent a potential global crisis. However, quantifiable risk factors for distinct PASC and their biological associations are poorly resolved. He discussed the ISB/Swedish INCOV study in which his group investigated several hundred COVID-19 patients and healthy donors from two cohorts using clinical data, patient-reported symptoms, viral load, anti-SARS-CoV-2 antibodies and autoantibodies, and multi-omic analyses of blood plasma and circulating immune cells. They resolved four factors that are significantly associated with PASC: SARS-CoV-2 RNAemia near the time of initial COVID-19 diagnosis, Epstein–Barr virus (EBV) viremia during acute disease, bystander activation of cytomegalovirus (CMV), and specific autoantibodies at convalescence. They also identified potential therapeutic strategies for a subset of patients suffering from PASC. Systematic analysis of symptom-associated immunological signatures revealed coordinated polarization of innate and adaptive immunity that was independently associated with acute infection severity and PASC. Their analyses suggest that the heterogeneous symptoms of long COVID arise from multiple independent sources. All human studies were performed following guidelines and protocols approved by the Institutional Review Board of the Institute of Systems Biology.

Sven Heinz, along with Samuel J. Roth and Christopher Benner (Dept. of Medicine, Division of Endocrinology and Metabolism, University of California San Diego), examined how transcriptome phenotyping identifies viruses that inhibit host cell transcription termination. Cells that recognize viral-pathogen-associated molecular patterns rapidly activate production of antiviral proteins. Some viruses, such as herpes simplex virus 1 and influenza A virus, subvert the host cell antiviral response in part by globally inhibiting transcription termination. This is caused by viral proteins that inhibit transcript cleavage and polyadenylation, leading to “reading through” of RNA polymerase II past the end of genes. The resulting unnaturally long readthrough transcripts, which include those coding for antiviral proteins, remain in the nucleus and are not translated into protein. Applying a bioinformatics approach to detecting readthrough transcription in public RNA-seq datasets from cells infected with various viruses, they found that Zika, Sindbis, and Rift Valley Fever virus (RVFV) inhibit transcription termination. They confirmed this finding for RVFV, where they showed that expressing RVFV NSs protein alone is sufficient to induce global transcriptional readthrough. Their findings suggest that genome-wide inhibition of transcription termination by viral proteins may represent a broader viral strategy to undermine the host cell antiviral response. No animal or human studies were performed.

Ryan Jeep ^1^, along with Christian Sanders ^1^, Megan Gish ^1^, Lillian Huang ^1^, and Chaoping Chen ^1^ (^1^ Department of Biochemistry and Molecular Biology, Colorado State University, Fort Collins, CO, USA), discussed their research on HIV protease inhibitor (PI) resistance. PI resistance compromises treatment efficacy and prognosis of combination antiretroviral therapy. The currently available PIs target the active site of mature protease (PR); however, resistance-associated mutations (RAMs) identified in patients experiencing drug resistance map to various regions of protease. When introduced into *E. coli* for protein purification and in vitro characterization, many RAMs do not display drug resistance at the magnitudes observed in patients. They previously reported that precursor autoprocessing is context-dependent, as its activity and outcomes can be modulated by sequences upstream of p6*-PR. Notably, the mature PRs released from select fusion precursors were self-degradation prone, resembling those purified from *E. coli*. In contrast, precursors with the 26aa maltose-binding protein signal peptide (SigP) at the N-terminus released mature PRs resistant to self-degradation resembling those found in viral particles. Therefore, SigP-containing fusion precursors likely represent a more biologically relevant “context” and are expected to manifest the clinically observed drug resistance. To investigate this context, they engineered a panel of fusion precursor constructs containing either the wild-type or an indinavir-resistance-associated double mutation (77I82T) in the PR region. Both wild-type and 77I82T mutant precursors showed similar responses to PI when released from constructs without SigP. However, the 77I82T mutant displayed a significant resistance profile when the N-terminal SigP was present in the precursors, which matches their viral infectivity analysis. These results support their context-dependent hypothesis and suggest that SigP-containing precursors better recapitulate PI response observed in a viral setting. This platform provides a unique insight for studying drug resistance. No animal or human studies were performed.

Jeffrey Kim (Department of Microbiology, Immunology, and Pathology, College of Veterinary Medicine and Biomedical Sciences, Colorado State University, Fort Collins, CO, USA) provided an update on combination antiretroviral therapy treatment for feline immunodeficiency virus (FIV). FIV is a naturally occurring retrovirus that causes progressive immune dysfunction in cats. Although effective treatments, including a combination antiretroviral therapy (cART), have been developed for human immunodeficiency virus, there is no therapy for FIV. This study aimed to assess a cART of Doulutegravir, Tenofovir, and Emtricitabine as a treatment for FIV in domestic cats. Eighteen 6- to 10-month-old cats were used with female intact and male neutered cats equally distributed among cART, placebo, and control groups. He inoculated twelve cats with FIV in addition to six naïve cats as controls and quantified the blood viral and proviral loads via digital droplet PCR pre-FIV inoculation and in weeks 0–24. Beginning in week 4, cats were injected with cART (n = 6) or a 15% kleptose placebo (n = 6), the cART vehicle, daily for the FIV-infected cats. Infection peaked at week 2 with mean viral loads of 6.43 × 10^6^ and 12.4 × 10^7^ copies/mL among cART and placebo cats, respectively; mean proviral loads were 5.43 × 10^4^ and 4.94 × 10^4^ copies/10^6^ cells among cART and placebo cats, respectively. Viral and proviral loads steadily declined afterwards and were not statistically significant. Hematologic analysis demonstrated marked neutropenia in placebo cats compared to cART (p = 0.05), with the lowest at week 6 with mean absolute values/uL of 651 and 1340, respectively. The mean neutrophil values in cART cats normalized by week 16, but placebo cats remained neutropenic. No clinical signs associated with neutropenia were observed. These results suggest that this cART protocol does not reduce FIV viral and proviral loads, but may impact the hematologic impacts of FIV infection. All animal studies were performed following guidelines and protocols approved by the Institutional Animal Care and Use Committee of Colorado State University.

Mani Kuan ^1^, L. Caruso ^2^, A. Zavala ^1^, P. Rana ^1^, J. O’Dowd ^1^, I. Tempera ^2^, and E. Fortunato E ^1^ (Department of Biological Sciences, University of Idaho ^1^ and The Wistar Institute ^2^) discussed how HCMV pp71 interaction with host DNA leads to downregulation of the basement membrane protein Nidogen 1. Nidogen 1 (NID1) is an important basement membrane protein. Their previous work found that HCMV infection rapidly induced chromosome 1 breaks and downregulated expression of NID1 both at the transcriptional and post-translational levels. NID1 downregulation promoted HCMV dissemination. They determined several viral proteins and some of their mechanisms involved in regulating NID1. Here, they investigated gene expression of NID1. Since regulation started by 24 hpi, they screened the most prominent viral proteins present at this timepoint, including incoming virus tegument and rapidly expressed immediate early (IE) proteins. They screened pp65, pp71, UL35, IE1, and IE2. They used an adenovirus (Ad) expression system to introduce each of these HCMV proteins into human foreskin fibroblasts. Using RT-qPCR, they found that pp71 downregulated NID1 gene transcription. Surprisingly, WF28-71, a fibroblast clone that expresses small amounts of pp71, suppressed NID1 transcript levels as efficiently as during HCMV infection and, therefore, resulted in a dramatically reduced steady-state (ss) level of NID1 protein. CCCTC-binding factor (CTCF) is a transcription factor that contributes with various regulatory roles in gene expression. There are CTCF binding sites located directly adjacent to the chromosome 1 break sites and the NID1 promoter. They suspected that pp71 might be interacting with CTCF to influence NID1 transcription. Chromatin immunoprecipitation found both pp71 and CTCF bound at these sites during HCMV infection. Knockdown CTCF fibroblasts infected with Ad-pp71 recovered the NID1 transcription and ss level of NID1 protein. pp71 is a key player in HCMV’s efforts to eliminate cellular NID1 and affects this paradigm via direct binding to the host cell genomic DNA and interaction with CTCF. No animal or human studies were performed.

Gabriela Samayoa Reyes (University of Colorado, Anschutz Medical Campus, Denver, CO) presented characterization of EBV in saliva of HIV+ mothers in a Kenyan population and presented evidence of mixed EBV infections and both methylated and unmethylated viral genomes. HIV infection is associated with EBV shedding in saliva, suggesting an increased risk of EBV transmission to infants born to mothers with HIV. She investigated the following questions: (i) Is HIV status a risk factor for EBV shedding in postpartum mothers? (ii) Is there a difference in EBV strains shed between HIV- positive (HIV+) and HIV-negative (HIV−) individuals? (iii) Is the viral DNA detected by qPCR representative of cell-associated infection or attributable to virions? Samples were from a cohort study in which HIV+ and HIV− pregnant women from Western Kenya were followed through delivery and the postpartum period. DNA was extracted from saliva and EBV load was measured by qPCR. She observed a significant relationship between EBV shedding and HIV status; HIV− mothers had a greater proportion of detectable EBV in their saliva compared to HIV+ mothers (p-value: 0.005). Of the mothers that were EBV shedders, those with HIV had significantly greater log EBV copies/mL of saliva (p-value: 0.015). Overall, she found that 47.3% of the EBV-positive samples were infected with EBV type 1, 23% with EBV type 2, and 24.3% were co-infected with no difference in frequency of EBV type attributable to the HIV− or HIV+ mothers. She analyzed the EBV genome methylation patterns using bisulfite sequencing to assess whether they were detecting cell-associated DNA (methylated) vs. virion-derived DNA (unmethylated). Interestingly, she observed two major groups that could be segregated based on methylation patterns and viral load. One group had high methylation and low viral load; the other was an unmethylated group associated with a higher EBV viral load, indicative of virion shedding. The presence of methylation indicates that a variable proportion of EBV DNA in saliva derives from cellular sources. In summary, HIV+ mothers that shed EBV have a higher viral load compared to HIV− mothers. All human studies were performed following guidelines and protocols approved by the Institutional Review Board of the University of Colorado, Anschutz Medical Campus.

